# Supplementary material for: A hydro-topological strategy enables self-regulating biofilms for sustainable wastewater treatment
Source: Nat Commun. 2026 Mar 13;17:3878. doi: 10.1038/s41467-026-70682-y (PMC13125250; doi:10.1038/s41467-026-70682-y)
Supplement: Supplementary file 1 — Supplementary Information [file 41467_2026_70682_MOESM1_ESM.pdf]

# Supplementary Information

## A hydro-topological strategy enables self-regulating biofilms for sustainable wastewater treatment

Yong Fang [1], Zhiqiang Zhang [2], Boru Xue [3], Ying Liu [1*], Kuichuan Sheng [1*]

[1] College of Biosystems Engineering and Food Science, Zhejiang University, Hangzhou, 310058, China

[2] Hangzhou Tao of Water Technology Co., Ltd, Room C117, Xiyuan Road 10, Xihu District, Hangzhou, 310000, China

[3] School of Mining Engineering, University of Science and Technology Liaoning, Anshan 114051, China

[*] Corresponding authors:

E-mail address: liuyingzju@zju.edu.cn (Ying Liu), kcsheng@zju.edu.cn (Kuichuan Sheng).

**Table of Contents:**

Supplementary Equations 1–10 Pages 3–8

Supplementary Notes 1–7 Pages 9–20

Supplementary Tables 1–5 Pages 21–25

Supplementary Figures 1–13 Pages 26–38

Supplementary References Pages 39–40

**Supplementary Equations | Analytical parameter calculations and procedures**

This note details the protocols for key analytical measurements and calculations performed in this study.

**1.1 Determination of carrier number, bulk density, and structural porosity**

**1.1.1 Determination of carrier number**

The total number of carriers in each reactor was determined as follows:

1. Clean and dry carriers was thoroughly mixed and placed into a 20-L graduated container.
2. The bulk volume of carriers was then calibrated again by subdividing into ten equal portions using a 2-L graduated container.
3. Three portions were selected at random. The number of carriers in each 2-L portion was counted manually, and then the number of per liter was calculated ($N_p$).
4. The total number of carriers ($N_t$) in the reactor was calculated using the Equation (1):

$$N_t = N_p \times V_r \times P_r \quad (1)$$

where $V_r$ is the working liquid volumes of reactor, $P_r$ is the corresponding volumetric packing ratio of carriers. In this study, the working liquid volumes of reactor were 150 L (anoxic) and 250 L (aerobic), the designed corresponding volumetric packing ratios were 16% and 30%.

Based on this method, the total numbers of V-carriers added to the anoxic and aerobic reactors were determined to be 24,000 and 75,000, respectively.

**1.1.2 Determination of carrier bulk density**

The bulk density of the clean, dry carriers was determined as follows:

1. Clean and dry carriers were thoroughly mixed and placed into a standard 1 $m^3$ container.

2. The container was filled, and the mass of carriers ($M_i$, kg) was measured. This procedure was repeated for five replicate containers.

The carrier's bulk density (kg m$^{-3}$) was calculated using Equation (2):

$$Bulk\ density = \frac{\sum_{i=1}^{i=5} M_i}{5} \tag{2}$$

**1.1.3 Determination of carrier structural porosity**

The structural porosity (i.e., the void volume fraction within the carrier's overall envelope) of the carrier bed was determined as follows:

1. Clean and dry carriers were thoroughly mixed and placed into a 20-L graduated container ($V_{20}$).
2. The container was filled with water to the 20-L mark. The carriers were then removed using a strainer, allowing excess water to drain back into the container. The volume of the remaining water ($V_i$, L) was measured.
3. This procedure was repeated for five replicates.

The carrier's structural porosity (%), defined as the void fraction within the bulk carrier volume, was calculated using Equation (3):

$$Structural\ porosity = \frac{\sum_{i=1}^{i=5} V_i}{5 \times V_{20}} \times 100\% \tag{3}$$

**1.2 Analysis of suspended and volatile suspended solids**

The total mass of suspended solids (SS) and volatile suspended solids (VSS) attributable to the biofilm in the reactor was determined to quantify the biomass inventory. The concentrations were then expressed per unit volume of the reactor.

**1.2.1 SS measurement:**

1. A sample of $\lambda$ carriers ($\lambda > 5$) was retrieved from the reactor. Suspended sludge was gently

rinsed from the carrier surfaces using a slow stream of water.

2. The carriers were dried in an oven at 105 °C until a constant weight ($M_1$) was achieved.
3. The carriers were then subjected to a rigorous cleaning procedure: they were soaked in a 10% (w/w) NaOH solution and treated with bath sonication and magnetic stirring (120 rpm) at 60 °C for 12 hours to dissolve the biofilm.
4. After cleaning, the carriers were thoroughly rinsed with tap water and dried again at 105 °C to a constant weight ($M_2$).
5. The SS concentration (mg L$^{-1}$) in the reactor was calculated using Equation (4):

$$SS = \frac{(M_1 - M_2) \times (\beta - \lambda)}{\lambda \times V_r} \tag{4}$$

where $\beta$ is the total number of carriers in the reactor before sampling, and $V_r$ is the working volume of the reactor (L). For each subsequent sampling event, the value of $\beta$ was updated to ($\beta - \lambda$) from the previous sampling.

**1.2.2 VSS measurement:**

1. A sample of $\lambda$ carriers ($\lambda > 5$) was retrieved from the reactor, and suspended sludge was gently rinsed off.
2. Biofilm was detached from the carriers by soaking in a 10% (w/w) NaOH solution with ultrasonic assistance.
3. The resulting suspension was filtered through a pre-dried, pre-weighed qualitative filter paper. The filter paper with the collected biofilm was dried at 105 °C to a constant weight ($M_1$). The constant weight of the pre-dried filter paper alone was $M_2$.
4. The dried filter paper with biofilm was then ignited in a muffle furnace at 550 °C for 2 hours. After cooling in a desiccator to room temperature, the final weight ($M_3$) was recorded.

5. The VSS concentration (mg L$^{-1}$) in the reactor was calculated using Equation (5):

$$VSS = \frac{(M_1 - M_2 - M_3) \times (\beta - \lambda)}{\lambda \times V_r} \tag{5}$$

where $\beta$ and $V_r$ are defined as in Equation (4).

### 1.3 Calculation of process performance metrics

#### 1.3.1 Nutrient removal efficiency

The removal efficiency (%) for a given nutrient (ammonia, $NH_4^+$-N; total inorganic nitrogen, TIN; or soluble chemical oxygen demand, sCOD) was calculated using Equation (6):

$$Removal\ Efficiency = \frac{N_{inf} - N_{eff}}{N_{inf}} \times 100\% \tag{6}$$

where $N_{inf}$ and $N_{eff}$ are the influent and effluent concentrations (mg L$^{-1}$) of the nutrient, respectively. Specifically, this yields the ammonia removal efficiency (ARE), TIN removal efficiency (NRE), and sCOD removal efficiency (CRE).

#### 1.3.2 Specific nutrient removal rate

The nutrient removal rate (g N g VSS$^{-1}$ day$^{-1}$ or g sCOD g VSS $^{-1}$ day$^{-1}$), normalized to the biofilm biomass (VSS), was calculated using Equation (7):

$$Removal\ Rate = \frac{24 \times V_{tot} \times \left(N_{inf} - N_{eff}\right)}{HRT \times VSS \times V_r} \tag{7}$$

where 24 is a factor converting hours to days; $V_{tot}$ is the total working volume of the bioreactor system (400 L); $N_{inf}$ and $N_{eff}$ are the influent and effluent concentrations (mg L$^{-1}$) of the nutrient, respectively; $HRT$ is the hydraulic retention time (hours); $VSS$ is the volatile suspended solids concentration calculated from Equation (5) (mg L$^{-1}$); and $V_r$ is the working volume of the specific reactor (anoxic or aerobic) from which the biomass sample was taken (L). This calculation yields the ammonia removal rate (ARR), TIN removal rate (NRR), or sCOD removal rate (CRR).

### 1.3.3 Ex situ nitrifying potential

The specific nitrifying potential of the biofilm, expressed as the ammonia oxidation rate per unit of biomass (g N g VSS$^{-1}$ day$^{-1}$), was determined from batch assays and calculated using Equation (8):

$$Nitrifying\ Potential = \frac{1440 \times (N_{init} - N_{fnl}) \times V_{batch}}{t_d} \times \frac{\beta}{300 \times VSS \times V_r} \quad (8)$$

where 1440 is a factor converting minutes to days; $N_{init}$ and $N_{fnl}$ are the initial and final $NH_4^+$-N concentrations (mg L$^{-1}$) in the batch test, with $N_{fnl}$ required to be > 15 mg L$^{-1}$ to avoid substrate limitation; $V_{batch}$ is the volume of the batch reactor (3 L); $t_d$ is the duration of the batch test (minutes); 300 is the number of carriers used in the batch test; $VSS$ is the VSS concentration in the aerobic reactor at the time of sampling; $V_r$ is the working volume of the aerobic reactor (250 L); and $\beta$ is the total number of carriers in the aerobic reactor.

### 1.3.4 Volumetric nitrifying rate (VNR)

The volumetric nitrifying rate of the aerobic reactor, expressed as the ammonia oxidation rate per unit of aerobic reactor working volume (g N m$^{-3}$ day$^{-1}$), was calculated using Equation (9):

$$VNR = R_s \times VSS \quad (9)$$

where $R_s$ is the in situ or ex situ specific nitrifying rate (g N g VSS$^{-1}$ day$^{-1}$), calculated from Equation (7) or (8) for in situ or ex situ conditions, respectively; $VSS$ is the VSS concentration in aerobic reactor, representing the total biofilm biomass per unit reactor volume (g VSS m$^{-3}$), calculated from Equation (5).

### 1.4 Biofilm thickness measurement

Biofilm thickness was measured directly on carriers using light microscopy.

1. For each measurement, five carriers were retrieved from the reactor.

2. Immediately after retrieval, the biofilm on the carriers was examined in situ using an industrial light microscope (GP-304K, China) under a consistent magnification.

3. Thickness ($T_i$, μm) was recorded at multiple predefined locations on each carrier. After measurement, the carriers were returned to the reactor. Data are presented as the mean ± standard deviation, and calculated using Equation (10):

$$Thickness = \frac{\sum_{i=1}^{i=5} T_i}{5} \tag{10}$$

**Supplementary Note 1 | Interpretation of the volatile suspended solids to suspended solids (VSS/SS) ratio**

The ratio of volatile suspended solids (VSS) to suspended solids (SS) is a critical parameter for assessing the physiological state and accumulation tendency of a biofilm. The concentrations of SS and VSS were determined using standard gravimetric methods, as detailed in Supplementary Equations 4 and 5.

- Suspended Solids (SS) represents the total dry mass of solids retained on a carrier, encompassing both organic and inorganic components. It is measured as the mass of residue after evaporation and drying at 105 °C.
- Volatile Suspended Solids (VSS) is the fraction of SS that is lost upon ignition at 550 °C, representing the organic matter, which primarily consists of active microbial biomass and extracellular polymeric substances.

Therefore, the VSS/SS ratio serves as a key indicator of biofilm composition and health:

- A high (e.g., > 0.7) and stable VSS/SS ratio indicates a biofilm dominated by active biomass with minimal accumulation of inorganic precipitates (scaling), signifying a healthy, metabolically active state conducive to efficient mass transfer.
- A low or declining VSS/SS ratio signals the accumulation of inorganic minerals (e.g., phosphates, carbonates) within the biofilm matrix. This increases the SS component without contributing to metabolic activity, leading to increased biofilm density, impaired mass transfer, and ultimately, clogging. In this study, carriers prone to clogging (e.g., K5, Mutagbiochip) exhibited a significant decline in VSS/SS ratio over time, correlating with performance failure (see Supplementary Table 1).

Thus, monitoring the VSS/SS ratio provides vital diagnostic information on the functional state of the biofilm and the long-term operational stability of the carrier system.

**Supplementary Note 2 | Biofilm Establishment and System Startup Protocol**

This note details the optimized startup protocol referenced in the main text. The primary objective was to rapidly establish a stable biofilm, with a specific focus on enriching autotrophic nitrifiers, to achieve the target treatment performance in the shortest possible time.

**1. Initial Setup and Inoculation**

As described in the main text, clean V-carriers were added to the reactors and fluidized for uniform distribution. Biofilm cultivation was initiated using activated sludge (MLSS = 3,512 mg $L^{-1}$) from the Chengxi WWTP (Hangzhou, China).

**2. Optimized Sludge Dosing Strategy**

The protocol of adding 50 L of activated sludge twice daily during the first three days was determined through a systematic comparison of strategies, with the time to achieve stable operation at the target HRT as the key metric:

| Dosing Strategy | Time to Reach Target HRT |
|---|---|
| No inoculation (natural colonization) | 21 days |
| Once-daily dosing (50 L) | 14 days |
| Twice-daily dosing (50 L per dose) | 10 days |
| Thrice-daily dosing (50 L per dose) | 9 days |

**3. Rationale for Selected Protocol**

The twice-daily protocol was selected as it offered an optimal balance, significantly reducing the startup time from 21 to 10 days compared to natural colonization, while remaining more operationally practical than thrice-daily dosing.

**4. Underlying Mechanism and HRT Adjustment**

This approach maintains a substantial initial microbial population. The subsequent formation

of an initial heterotrophic biofilm layer enhances extracellular polymeric substances (EPS) production, which promotes biomass-carrier adhesion and accelerates the development of the nitrifying biofilm[1]. Following the 3-day inoculation period, the HRT was progressively reduced by increasing the inflow rate until stable nutrient removal was achieved, marking the start of the long-term experimental phase.

**Supplementary Note 3 | Elemental analysis via ICP-MS**

The elemental composition (P, Ca, Mg) of the biofilm was analyzed to assess inorganic precipitation (scaling).

1. Biofilm was harvested from 100 carriers as described in the VSS method (Steps 1-2).
2. The collected biofilm was dried at 105 °C to a constant weight.
3. Approximately 0.10 g of the dried material was digested in a PTFE vessel with 4 mL of aqua regia ($HNO_3$:HCl, 3:1 v/v) and 1 mL of HF.
4. Digestion was performed using a temperature program: ramped at 2 °C $min^{-1}$ to 120 °C (hold 3 min), then to 180 °C (hold 30 min), and finally to 200 °C (hold 15 min).
5. The digestate was cooled, diluted to 50 mL with ultrapure water, and filtered through a 0.22-μm membrane.
6. The filtrate was further diluted 500-fold prior to analysis by inductively coupled plasma mass spectrometry (ICP-MS, iCAP RQ, Thermo Fisher Scientific). Results were calculated based on dilution factors and sample mass, expressed as mg element per g of dried biofilm (mg $g^{-1}$).

**Supplementary Note 4 | Processing and analysis of 16S rRNA amplicon sequencing data**

**1. Sample preparation and sequencing**

To investigate microbial community dynamics, genomic DNA was extracted from biofilm biomass sampled on days 45, 156, 307, and 455 (n = 3 biological replicates per time point). The V3–V4 hypervariable regions of the bacterial 16S rRNA gene were amplified using primers 341F (5′-CCTAYGGGRBGCASCAG-3′) and 806R (5′-GGACTACNNGGGTATCTAAT-3′)[2]. Polymerase chain reaction (PCR) was performed on a T100 Thermal Cycler (Bio-Rad, USA) under the following conditions: initial denaturation at 95 °C for 3 min; 27 cycles of denaturation at 95 °C for 30 s, annealing at 55 °C for 30 s, and extension at 72 °C for 45 s; followed by a final extension at 72 °C for 10 min. PCR products were verified by 2% agarose gel electrophoresis, purified using a PCR Clean-Up Kit (YuHua, China), and quantified. Equimolar amounts of purified amplicons were pooled for paired-end sequencing (2 × 300 bp) on an Illumina NextSeq 2000 platform (Illumina, USA) at Majorbio Bio-Pharm Technology Co., Ltd. (Shanghai, China), following manufacturer's protocols.

**2. Bioinformatic processing**

Raw sequencing data were processed using the QIIME2 pipeline (version 2024.2). Briefly, sequence quality control, adapter trimming, and quality filtering were performed with fastp (version 0.23.4). Paired-end reads were merged using FLASH (version 1.2.11), and denoising was conducted with the DADA2 plugin to resolve amplicon sequence variants (ASVs). To account for uneven sequencing depth, all samples were rarefied to 10,252 sequences per sample for downstream analysis, which yielded an average Good’s coverage of 97.90%, indicating sufficient sequencing depth. Taxonomic classification of ASVs was assigned using a pre-trained

naive Bayes classifier against the SILVA 16S rRNA gene database (release 138). All bioinformatic analyses were performed on the Majorbio Cloud Platform (https://cloud.majorbio.com).

**Supplementary Note 5 | Fluorescence in situ hybridization (FISH) protocol for biofilm analysis**

**1. Sample fixation and embedding**

Fresh V-carrier samples were collected from the aerobic reactor and gently rinsed with phosphate-buffered saline (PBS) to remove loosely attached suspended sludge. Samples were immediately fixed in 4% (w/v) paraformaldehyde at 4 °C for 1.5 h. Fixed samples were dehydrated through a graded ethanol series (50%, 70%, 80%, 95%, and 100%), cleared in xylene, and embedded in paraffin using standard histological protocols.

**2. Section preparation and pretreatment**

Paraffin-embedded biofilm samples were sectioned at 5 μm thickness using a rotary microtome (KD-P, KEDEE, China). Sections were mounted on glass slides and baked at 65 °C for 2 h to improve adhesion. Deparaffinization was performed by sequential immersion in: xylene I (15 min), xylene II (15 min), xylene III (15 min), anhydrous ethanol I (5 min), anhydrous ethanol II (5 min), 85% ethanol (5 min), 75% ethanol (5 min), and finally rinsed in diethylpyrocarbonate (DEPC)-treated water. Tissue sections were delineated with a hydrophobic barrier pen (WG1066-1, Gene Tech, China) and predigested with proteinase K (0.1 mg/mL, ST535, Beyotime, China) at 37 °C for 30 min, followed by three 5-min washes in PBS.

**3. Hybridization and detection**

Fluorescence in situ hybridization was performed using the following probes: NSO190 (5′-CGATCCCCTGCTTTTCTCC-3′, Alexa Fluor 488-labeled) targeting ammonia-oxidizing bacteria (AOB), and Ntspa712 (5′-CGCCTTCGCCACCGGCCTTCC-3′, Cy3-labeled)

targeting *Nitrospira* spp. (NOB)[3]. Sections were prehybridized at 37 °C for 1 h in hybridization buffer without probes, followed by overnight hybridization at 37 °C with probe cocktails (30% formamide, 0.9 M NaCl, 20 mM Tris-HCl, 0.01% SDS). Post-hybridization washes were performed in prewarmed SSC buffer (0.9 M NaCl, 20 mM Tris-HCl) at 37 °C for 30 min.

**4. Counterstaining and imaging**

Sections were counterstained with 4′,6-diamidino-2-phenylindole (DAPI, 1 μg/mL, D9542, Sigma, Germany) for 10 min at room temperature in the dark to visualize total cells, followed by three 5-min PBS washes. Slides were mounted using an anti-fade mounting medium (SBA-0100-01, SouthernBiotech, USA) and coverslipped. Confocal laser-scanning microscopy was performed using an Eclipse Ti microscope (Nikon, Japan) with appropriate filter sets for DAPI (excitation 405 nm), Alexa Fluor 488 (excitation 488 nm), and Cy3 (excitation 552 nm). Z-stack images were acquired at 0.5-μm intervals and processed using NIS-Elements software (Nikon).

**Supplementary Note 6 | Protocol for Live-Dead Cell Staining of Biofilms**

This protocol describes the procedure for staining and visualizing live and dead bacterial cells within a biofilm using the fluorescent nucleic acid stains SYTO 9 (Invitrogen, America) and propidium iodide (PI) (Solarbio, China), followed by confocal laser scanning microscopy (CLSM; ZEISS LSM 900, Germany) imaging.

1. Sample Preparation and Staining

Samples (e.g., biofilm-carriers) are used directly without the need for prior configuration or sterilization. Prepare a fresh staining working solution containing 5 μM SYTO 9 and 2 μg/mL PI in an appropriate buffer (PBS). Pipette 500 μL of the staining working solution to ensure it completely covers the sample surface. Incubate the sample in the dark at 37°C for 15 minutes to allow for sufficient dye penetration and binding. After incubation, gently rinse the sample twice with PBS buffer to remove any unbound dye.

2. Image Acquisition via CLSM

Imaging was performed using a 100× oil immersion objective. SYTO 9 (all cells) was excited at 495 nm with emission collected at 519 nm, while PI (dead cells) was excited at 305 nm with emission collected at 617 nm. For visualization, the SYTO 9 signal was pseudo-coloured blue to represent live cells, and the PI signal was shown in red for dead cells. Both 2D optical sections and z-stack series were acquired for high-resolution analysis at specific depths and 3D reconstruction of biofilm architecture, respectively.

**Supplementary Note 7 | Model Validation and Geometrical Boundary Conditions of the CFD Model**

**Model Validation**

The carrier geometry used in our simulations was modelled at a 1:1 scale (Supplementary Fig. 13). To validate the predictive accuracy of the CFD model under realistic flow conditions, we compared simulation results with the high-resolution experimental $\mu$PIV dataset from Campos Marín et al. (2017)[4], which provides quantified velocity fields within a 3D porous scaffold under controlled microfluidic conditions.

The validation benchmark was based on a polycaprolactone scaffold produced by additive manufacturing and analyzed using a 2D $\mu$PIV system. The scaffold pore size (0–1000 μm) is comparable to the characteristic dimensions of our MBBR carriers, establishing a relevant benchmark for validating the model's accuracy at the microscale.

Quantitative comparison between simulated and experimental velocity profiles showed close agreement, with a sum of squared errors (S) of $2.198\times10^{-3}$ and a sum of absolute differences (D) of $2.366\times10^{-3}$, yielding a relative error of 4.31%. This falls within the typical uncertainty range of $\mu$PIV systems (~10%), confirming the accuracy of our CFD model in capturing microscale flow behaviour essential for simulating hydraulic shear on biofilm carriers.

The consistency between CFD results and experimental benchmark validates the model's capability to resolve critical flow parameters at biofilm-relevant scales, providing a reliable foundation for analysing carrier hydrodynamics and self-cleaning mechanisms in wastewater treatment applications.

**Geometrical Boundary Conditions of the CFD Model**

The computational domain was designed to replicate the physical geometry of the MBBR system. Boundary conditions were assigned as follows:

**Inlet**: A velocity-inlet condition was applied with a uniform velocity of $U_X = 0.35$ m s$^{-1}$. This flow velocity was determined from multi-point measurements of the actual reactor and has been validated in other full-scale MBBR applications[5,6]. The fluid was modeled as water ($\rho = 997.561$ kg m$^{-3}$, $\mu = 8.8871\times10^{-4}$ Pa·s), and turbulence was introduced with an intensity of $I = 5\%$.

**Outlet**: An outflow condition was used to simulate fully developed flow without reverse flux.

**Walls**: The side, bottom, and MBBR external surfaces were treated as stationary no-slip walls, consistent with standard viscous flow assumptions[7].

These conditions ensured a physiologically realistic hydrodynamic environment, enabling accurate predictions of flow behavior and shear stress distributions critical to biofilm dynamics and nutrient transport.

**Supplementary Table 1 | Phosphorus, calcium, and magnesium content in biofilms from different carriers.**

| Time point | Carrier type | P (mg $g^{-1}$) | Ca (mg $g^{-1}$) | Mg (mg $g^{-1}$) |
|---|---|---|---|---|
| **Day 30** | V | 7.63 ± 0.03 | < 1.0 | < 1.0 |
| | Mutagbiochip | 9.37 ± 0.15 | < 1.0 | < 1.0 |
| | K3 | 23.68 ± 1.03 | < 1.0 | < 1.0 |
| | K5 | 32.74 ± 0.88 | 3.16 ± 0.13 | < 1.0 |
| | U | 21.14 ± 1.22 | 1.25 ± 0.03 | < 1.0 |
| **Day 150** | V | 25.06 ± 2.41 | 2.33 ± 0.08 | 1.55 ± 0.06 |
| | Mutagbiochip | 57.87 ± 1.92 | 21.42 ± 0.86 | 1.21 ± 0.04 |
| | K3 | 27.35 ± 1.82 | 2.44 ± 0.11 | 1.87 ± 0.05 |
| | K5 | 148.36 ± 3.11 | 54.31 ± 1.33 | 2.77 ± 0.51 |
| | U | 52.32 ± 2.55 | 11.91 ± 0.82 | 1.37 ± 0.12 |
| **Day 450** | V | 23.65 ± 1.12 | 2.33 ± 0.08 | 1.28 ± 0.04 |
| | Mutagbiochip | 93.64 ± 1.18 | 69.33 ± 0.74 | 2.25 ± 0.05 |
| | K3 | 26.27 ± 1.32 | 3.92 ± 0.17 | 1.21 ± 0.05 |
| | K5 | 205.19 ± 3.73 | 167.33 ± 5.46 | 7.89 ± 0.38 |
| | U | 102.49 ± 3.05 | 63.02 ± 1.47 | 4.46 ± 0.22 |

**Footnotes:**

All values represent mean ± standard deviation (n = 3 technical replicates). Values below the detection limit (< 1.0 mg $g^{-1}$) are indicated accordingly. Elemental content was determined by ICP-MS analysis of dried biofilm samples as described in Supplementary Note 3.

**Supplementary Table 2 | Microbial alpha diversity indices of V-carrier biofilms during long-term operation.**

| Sampling time | Observed ASVs (Sobs) | ACE index | Chao1 index | Shannon index | Simpson index (1-D) | Good's coverage |
|---|---|---|---|---|---|---|
| Day 45 | 776 | 809.54 | 799.77 | 4.66 | 0.963 | 0.998 |
| Day 156 | 798 | 820.71 | 809.62 | 5.23 | 0.984 | 0.999 |
| Day 307 | 885 | 933.26 | 925.22 | 5.36 | 0.986 | 0.998 |
| Day 455 | 871 | 891.72 | 880.17 | 4.99 | 0.972 | 0.999 |

**Footnotes:**

All samples were rarefied to 10,252 sequences per sample prior to diversity calculations. ACE, abundance-based coverage estimator; ASVs, amplicon sequence variants. Simpson index is presented as 1-D to facilitate interpretation (higher values indicate greater diversity).

**Supplementary Table 3 | Wastewater characteristics and effluent discharge standards.**

| Parameter | STE [a] | Raw wastewater [b] | Discharge standard [c] |
|---|---|---|---|
| **sCOD (mg $L^{-1}$)** | 274.1 ± 41.7 | 143.8 ± 23.4 | 30 |
| **$NH_4^+$-N (mg $L^{-1}$)** | 86.2 ± 11.5 | 45.9 ± 6.7 | 1.5 (3) [d] |
| **TIN (mg $L^{-1}$)** | 93.4 ± 14.3 | 50.7 ± 7.4 | 10 (12) [d] |
| **TP (mg $L^{-1}$)** | 17.5 ± 2.4 | 4.8 ± 1.7 | 0.3 |
| **SS (mg $L^{-1}$)** | 287.2 ± 45.3 | 168.5 ± 31.2 | < 10 |
| **pH** | 7.21 ± 0.09 | 7.27 ± 0.08 | 6–9 |
| **C/N ratio** | 2.9 ± 0.3 | 2.8 ± 0.3 | - |

**Footnotes:**

[a] STE, septic tank effluent collected from Shangkun Park (Hangzhou, China).

[b] Raw wastewater prepared by diluting STE with tap water to simulate typical influent concentrations. Data are presented as mean ± standard deviation (n = 520 independent replicates collected daily over the operational period).

[c] Discharge standard for major water pollutants from municipal wastewater treatment plants in Zhejiang Province, China (DB33/2169-2023).

[d] Values in parentheses indicate the standard applicable during the period from November 1 to March 31 of the following year.

Analytical methods: sCOD, $NH_4^+$-N, TIN, and TP were determined spectrophotometrically (GNST-900S, China) using Genesite® kits; SS was measured spectrophotometrically (GNST-900S-SS, China); pH was measured with a multiparameter sensor (HC2200, USA); C/N ratio was calculated from sCOD and TIN values.

**Supplementary Table 4 | Primers and thermal cycling conditions for qPCR analysis.**

| Target group (Gene) | Primer name | Sequence (5′→3′) | Thermal cycling conditions | Reference |
|---|---|---|---|---|
| **Total bacteria** (16S rRNA) | 1055f | ATG GCT GTC GTC AGC T | 95 °C for 3 min; 35 cycles of: | 8 |
| | 1392r | ACG GGC GGT GTG TAC | 95 °C for 30 s, 55 °C for 30 s, 72 °C for 60 s | 9 |
| **AOB** (beta-*Proteobacteria amoA*) | amoA-1f | GGG GTT TCT ACT GGT GGT | 94 °C for 10 min; 40 cycles of: | 10 |
| | amoA-2r | CCC CTC KGS AAA GCC TTC TTC | 94 °C for 15 s, 60 °C for 60 s, 72 °C for 60 s | |
| **AOB** (beta-*Proteobacteria*16S rRNA) | CTO-189f | GGA GRA AAG CAG GGG ATC G | 93 °C for 1 min; 35 cycles of: | 11 |
| | RT1r | CGT CCT CTC AGA CCA RCT ACT G | 92 °C for 30 s, 57 °C for 60 s, 68 °C for 45 s | 12 |
| **NOB** (*Nitrospira* spp. 16S rRNA) | Nspra-675f | GCG GTG AAA TGC GTA GAK ATC G | 94 °C for 5 min; 40 cycles of: | 13 |
| | Nspra-746r | TCA GCG TCA GRW AYG TTC CAG AG | 94 °C for 30 s, 65 °C for 30 s, 72 °C for 30 s | |
| **Archaea** (archaeal *amoA*) | Arch-amoAF | STA ATG GTC TGG CTT AGA CG | 94 °C for 5 min; 35 cycles of: | 13 |
| | Arch-amoAR | GCG GCC ATC CAT CTG TAT GT | 94 °C for 15 s, 65 °C for 15 s, 72 °C for 15 s | |

**Footnotes:**

Degenerate bases: R = A/G, S = G/C, K = G/T, W = A/T, Y = C/T.

AOB, ammonia-oxidizing bacteria; NOB, nitrite-oxidizing bacteria.

All reactions included a final extension at 72 °C for 7–10 min and a melt curve analysis.

**Supplementary Table 5 | Composition of synthetic wastewater for ex situ nitrifying potential assays.**

| Component | Chemical formula | Concentration | Unit |
|---|---|---|---|
| **Ammonium source** | | | |
| Ammonium chloride | $NH_4Cl$ | 0.38 | g $L^{-1}$ |
| **Macronutrients** | | | |
| Monopotassium phosphate | $KH_2PO_4$ | 0.025 | g $L^{-1}$ |
| Calcium chloride dihydrate | $CaCl_2·2H_2O$ | 0.20 | g $L^{-1}$ |
| Magnesium sulfate heptahydrate | $MgSO_4·7H_2O$ | 0.10 | g $L^{-1}$ |
| **Trace elements** | | | |
| Ferrous sulfate heptahydrate | $FeSO_4·7H_2O$ | 0.010 | g $L^{-1}$ |
| Zinc sulfate heptahydrate | $ZnSO_4·7H_2O$ | 0.005 | g $L^{-1}$ |
| Copper sulfate pentahydrate | $CuSO_4·5H_2O$ | 0.005 | g $L^{-1}$ |
| Manganese sulfate monohydrate | $MnSO_4·H_2O$ | 0.005 | g $L^{-1}$ |
| **Buffer** | | | |
| Sodium bicarbonate | $NaHCO_3$ | Dynamically supplied [a] | - |

**Footnotes:**

[a] Sodium bicarbonate solution (150 g $L^{-1}$) was continuously supplied at 0.1 mL $min^{-1}$ using a peristaltic pump to maintain pH at 7.5 during batch assays. All other components were prepared as a stock solution and added to the batch reactor at the beginning of the assay.

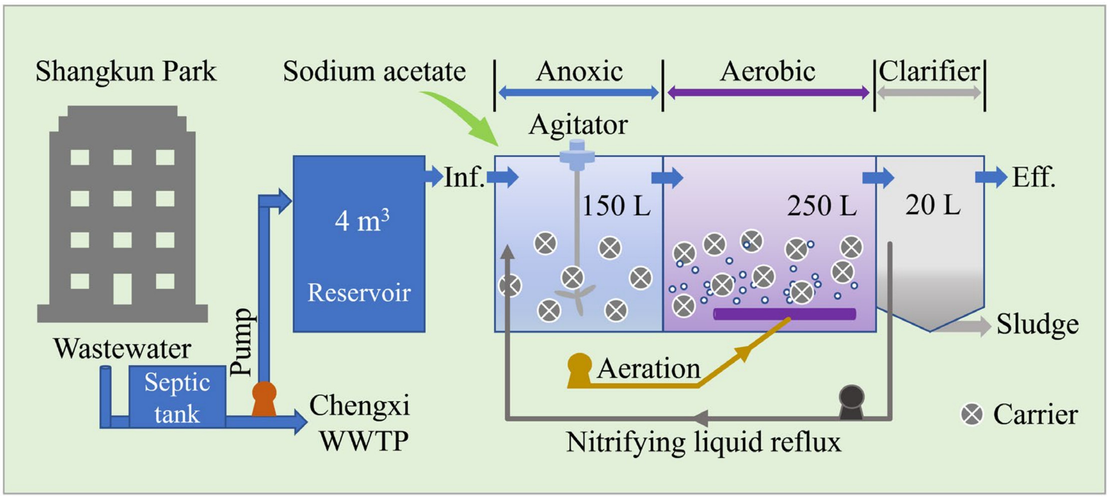


**Supplementary Fig. 1 | Schematic diagram of the anoxic/aerobic-moving bed biofilm reactor (A/O-MBBR) system.** Municipal wastewater from the septic tank of Shangkun Park was pumped into a 4-m$^3$ reservoir. Sodium acetate was supplemented as an external carbon source. The treatment train consisted of an anoxic reactor (150 L) equipped with a mechanical agitator and an aerobic reactor (250 L) with fine-bubble aeration, both packed with V-carriers, followed by a secondary clarifier (20 L). The system was operated in a pure biofilm mode without sludge recirculation. Nitrified liquor was recirculated from the clarifier to the anoxic zone. Eff., effluent.

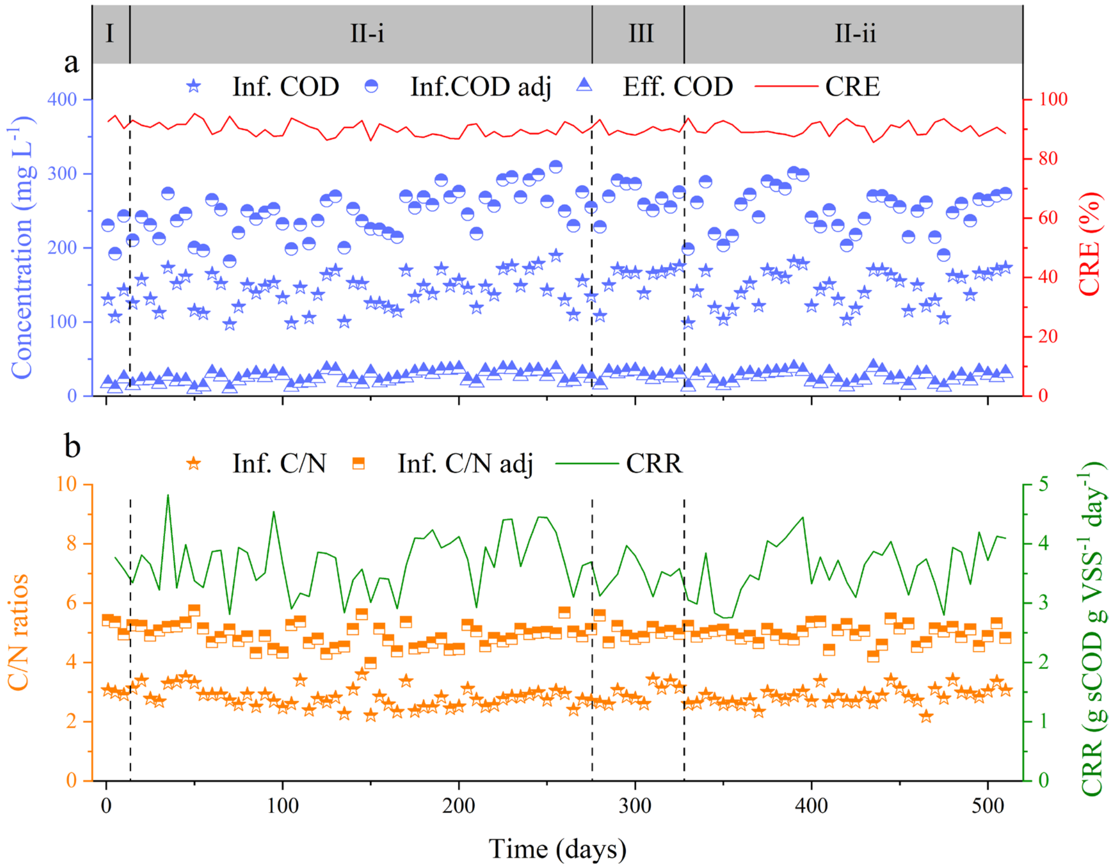


**Supplementary Fig. 2 | Long-term performance of organic matter removal in the A/O-MBBR system packed with V-carriers. a,** Temporal profiles of soluble chemical oxygen demand (sCOD) concentrations in the raw influent (Inf.), sodium acetate-adjusted influent (Inf. adj), final effluent (Eff.), and the corresponding sCOD removal efficiency (CRE). The operational phases (I, II-i, III, II-ii) are indicated at the top. **b,** Temporal profiles of the carbon-to-nitrogen (C/N) ratios of the raw influent (Inf. C/N) and sodium acetate-adjusted influent (Inf. C/N adj), alongside the specific sCOD removal rate (CRR). The shaded areas in **a** and **b** distinguish the operational phases based on hydraulic retention time and temperature: Phase I (start-up, days 1–10), Phase II-i (suitable temperature, days 11–280), Phase III (low temperature, days 281–324), and Phase II-ii (suitable temperature, days 325–512).

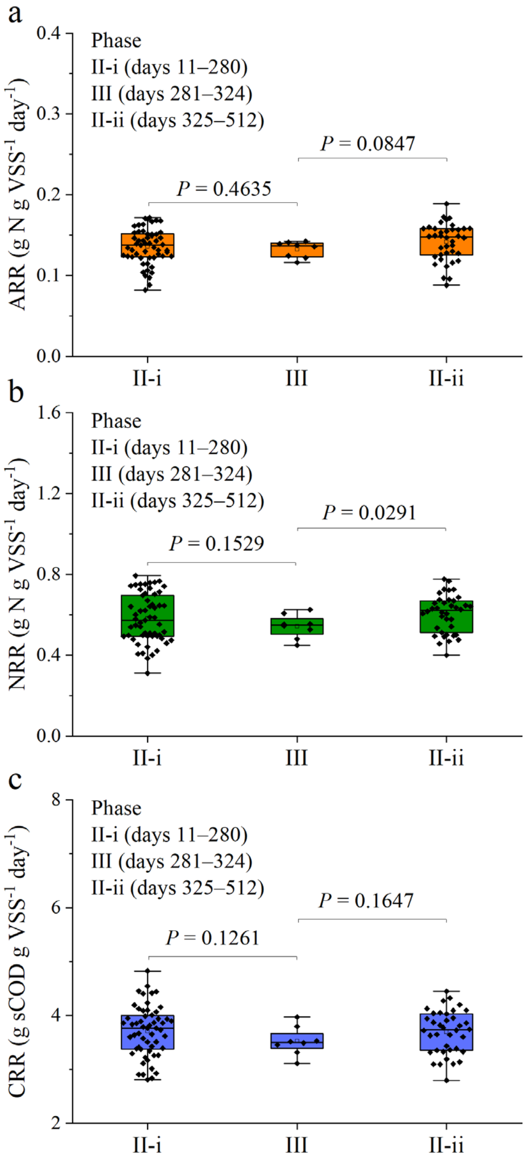


**Supplementary Fig. 3 | In situ nutrient removal rates in the V-carrier A/O-MBBR system under different operational phases. a,** Ammonia removal rate (ARR). **b,** Total inorganic nitrogen removal rate (NRR). **c,** Soluble chemical oxygen demand removal rate (CRR). Data are presented as box plots showing the median (center line), first and third quartiles (box limits), and 1.5× interquartile range (whiskers). The number of independent measurements (*n*) for each phase is as follows: Phase II-i (suitable temperature, n = 55), Phase III (low temperature, n = 8), and Phase II-ii (suitable temperature, n = 38). Statistical significance between phases was assessed using a two-tailed paired *t*-test; exact *P* values are reported on the graphs. Note the consistent and high removal rates maintained across all phases, including the low-temperature Phase III, demonstrating the robustness of the system.

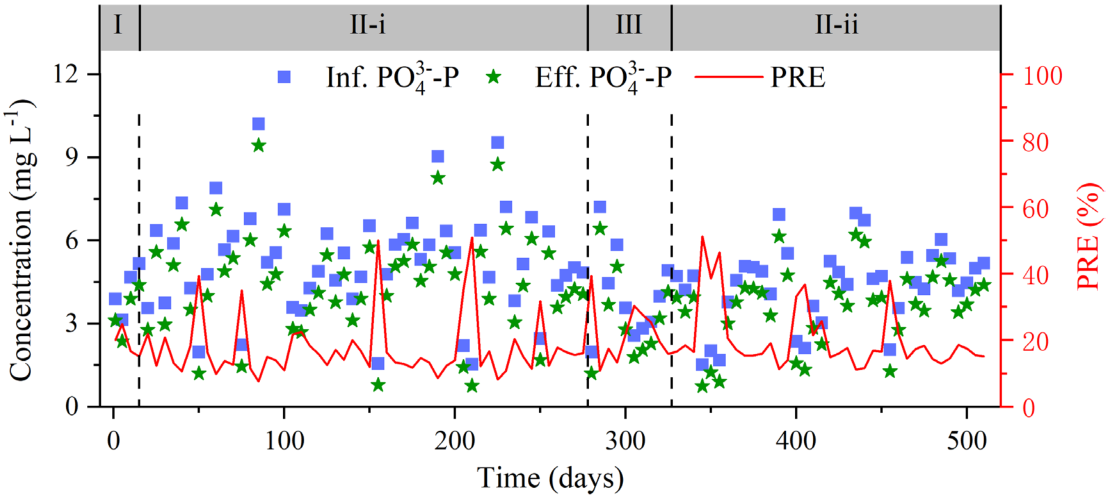


**Supplementary Fig. 4 | Phosphorus removal performance of the A/O-MBBR system packed with V-carriers during long-term operation.** Temporal profiles of influent (Inf. $PO_4^{3-}$-P, blue squares), effluent (Eff. $PO_4^{3-}$-P, green stars) orthophosphate concentrations (left y-axis), and phosphorus removal efficiency (PRE, red curve, right y-axis). Operational phases are indicated at the top: Phase I (start-up, days 1–10), Phase II-i (suitable temperature, days 11–280), Phase III (low temperature, days 281–324), and Phase II-ii (suitable temperature, days 325–512).

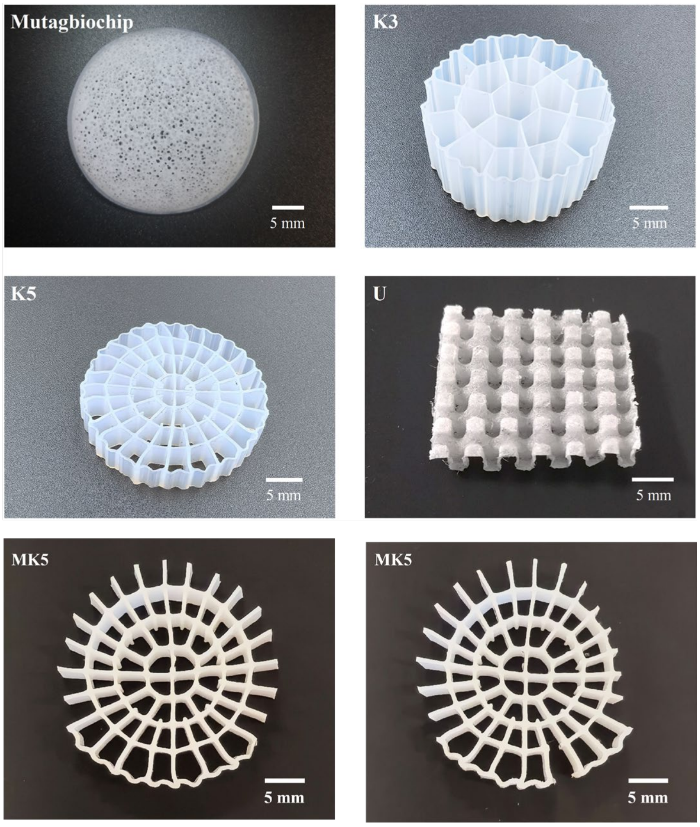


**Supplementary Fig. 5 | Structural overview and experimental setup of conventional carriers for comparative analysis.** Photographs of four conventional biofilm carriers used in parallel control systems: Mutagbiochip (Multi Umwelttechnologie AG, Germany), K3 and K5 (Veolia Water Technologies, Sweden), and U-carrier (Hangzhou Tao of Water Technology, China). Scale bars, 5 mm. Notably, the modified K5 carrier (MK5) was engineered by removing the outer grid section of standard K5 carriers to create hybrid structures with both open and enclosed sections. All carriers (except for MK5 carrier) were tested in independent A/O-MBBR systems with identical configuration and operational parameters as the V-carrier system (Supplementary Fig. 1). Systems were operated with volumetric packing ratios of 16% (anoxic) and 30% (aerobic), uniform aeration control, and identical mixing conditions. All reactors received the same regulated wastewater from a shared reservoir. Hydraulic retention time was managed according to Zhejiang Province discharge standards (Supplementary Table 3). The K5-based system included 1,000 MK5 carriers in the aerobic reactor to evaluate the effect of topological modification on biofilm development.

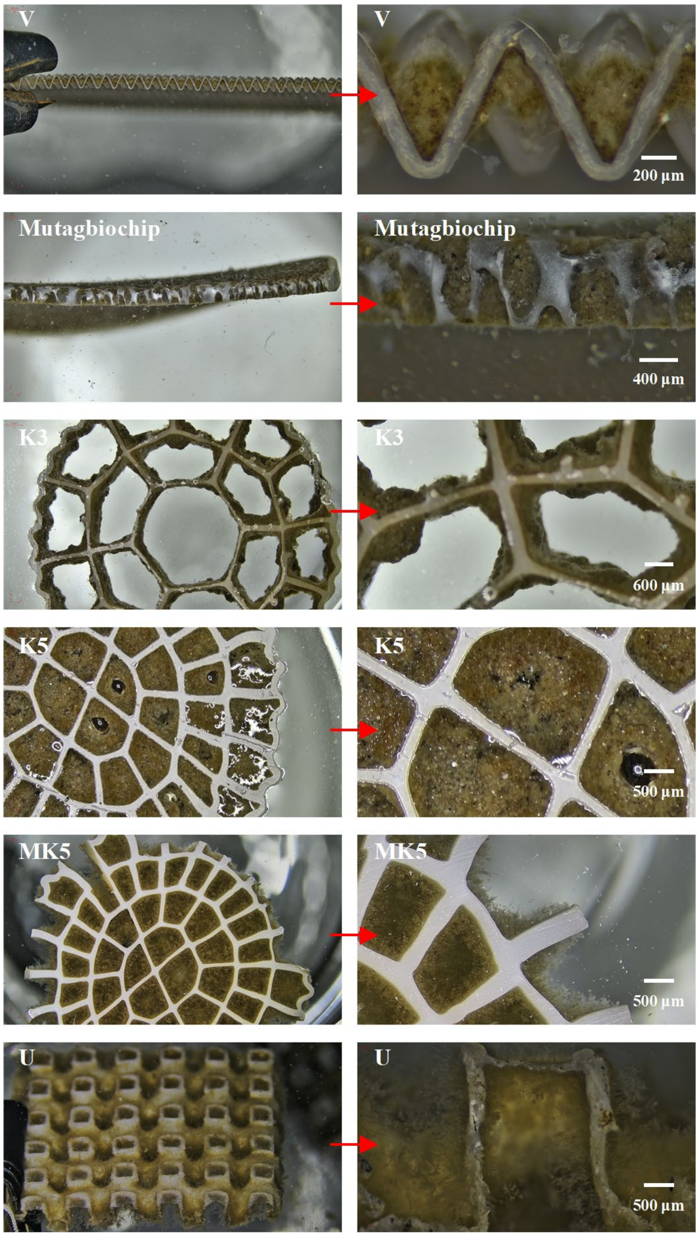


**Supplementary Fig. 6 | Comparative analysis of biofilm morphology on various carriers after 415 days of aerobic operation.** Light micrographs showing representative biofilm development on V, Mutagbiochip, K3, K5, modified K5 (MK5), and U-carriers. Left panels show low-magnification overviews of carrier structures with biofilm colonization; red arrows indicate locations selected for high-magnification analysis. Right panels show corresponding high-magnification views revealing detailed biofilm architecture, thickness, and surface morphology. Note the distinct biofilm distribution patterns influenced by carrier topology, including complete clogging in small-hollow carriers (K5, Mutagbiochip) versus uniform biofilm coverage in large-hollow carriers (K3). The MK5 carrier shows contrasting biofilm development between open-edge and protected-hollow sections, demonstrating the critical role of hydraulic exposure in biofilm self-cleaning.

a

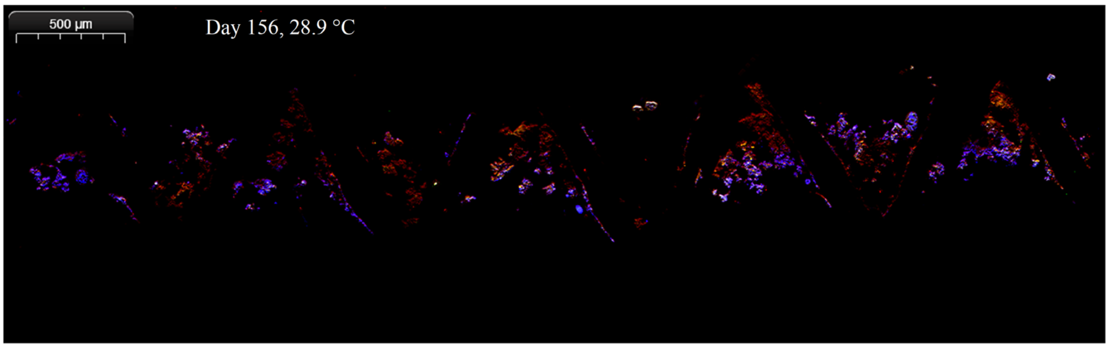


b

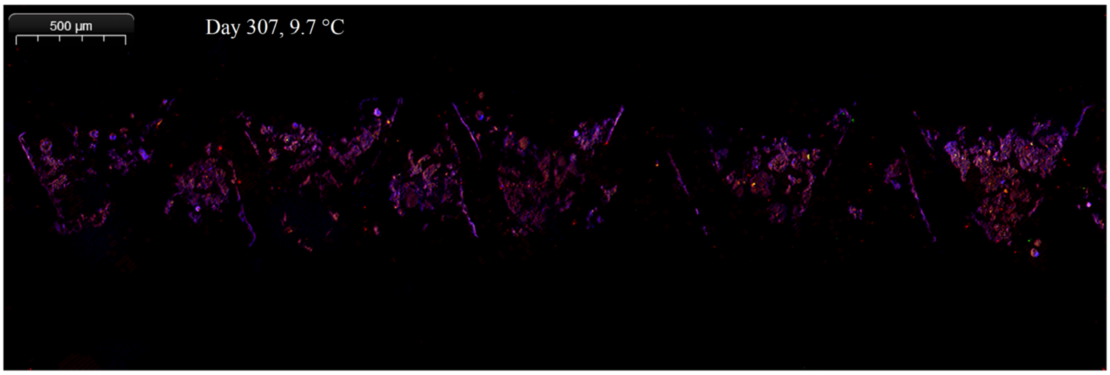


**Supplementary Fig. 7 | Temperature-dependent spatial reorganization of nitrifying bacteria in V-carrier biofilms revealed by FISH-CLSM. a,** Biofilm cross-section sampled on day 156 (summer conditions, 28.9 °C) showing robust co-localization of ammonia-oxidizing bacteria (AOB, green; targeting amoA gene) and nitrite-oxidizing bacteria (NOB, red; targeting Nitrospira 16S rRNA), with orange regions indicating spatial overlap. Representative of n = 6 independent experiments with similar results. **b,** Biofilm cross-section sampled on day 307 (winter conditions, 9.7 °C) showing reduction in AOB and NOB signal intensity and altered spatial distribution. Representative of n = 6 independent experiments with similar results. Total bacterial cells are stained with DAPI (blue). Scale bars: 500 µm (applies to both panels). Images represent maximum intensity projections of z-stacks acquired under identical CLSM settings. The temperature-driven shift from co-localized to segregated distributions demonstrates microbial adaptation strategies supporting functional resilience under seasonal variations.

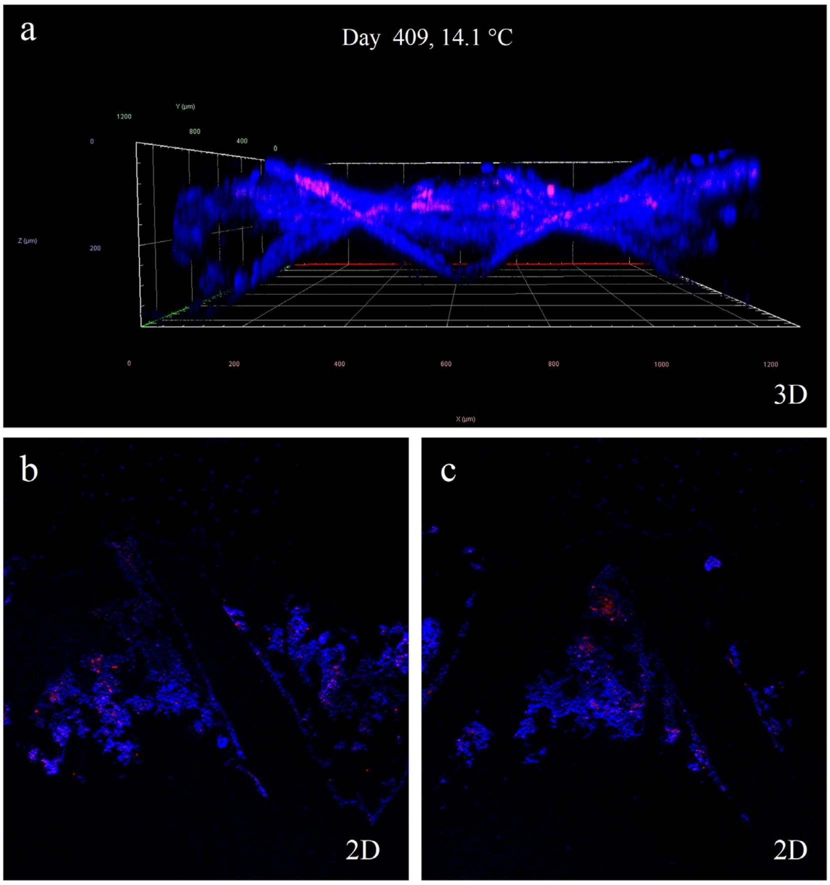


**Supplementary Fig. 8 | Evidence of cell lysis and self-renewal in V-carrier biofilms revealed by live-dead staining.** V-carrier biofilms were sampled after 409 days of operation and analyzed by confocal laser scanning microscopy (CLSM). **a,** Three-dimensional reconstruction showing the spatial distribution of live and dead cells. **b–c,** Representative two-dimensional optical sections at different focal planes. Cells were stained with SYTO 9 (excitation/emission: 495 nm / 519 nm) and propidium iodide (PI) (excitation/emission: 305 nm / 617 nm). For visualization, the SYTO 9 signal (all cells) is pseudo-coloured blue, and the PI signal (dead cells) is shown in red. The images reveal a higher proportion of dead cells (red) and lysing debris in the inner layers, illustrating a viability gradient from the interior to the bulk liquid. This provides direct visual evidence that internal voids serve as pathways for cellular material export, confirming an ongoing self-cleaning process.

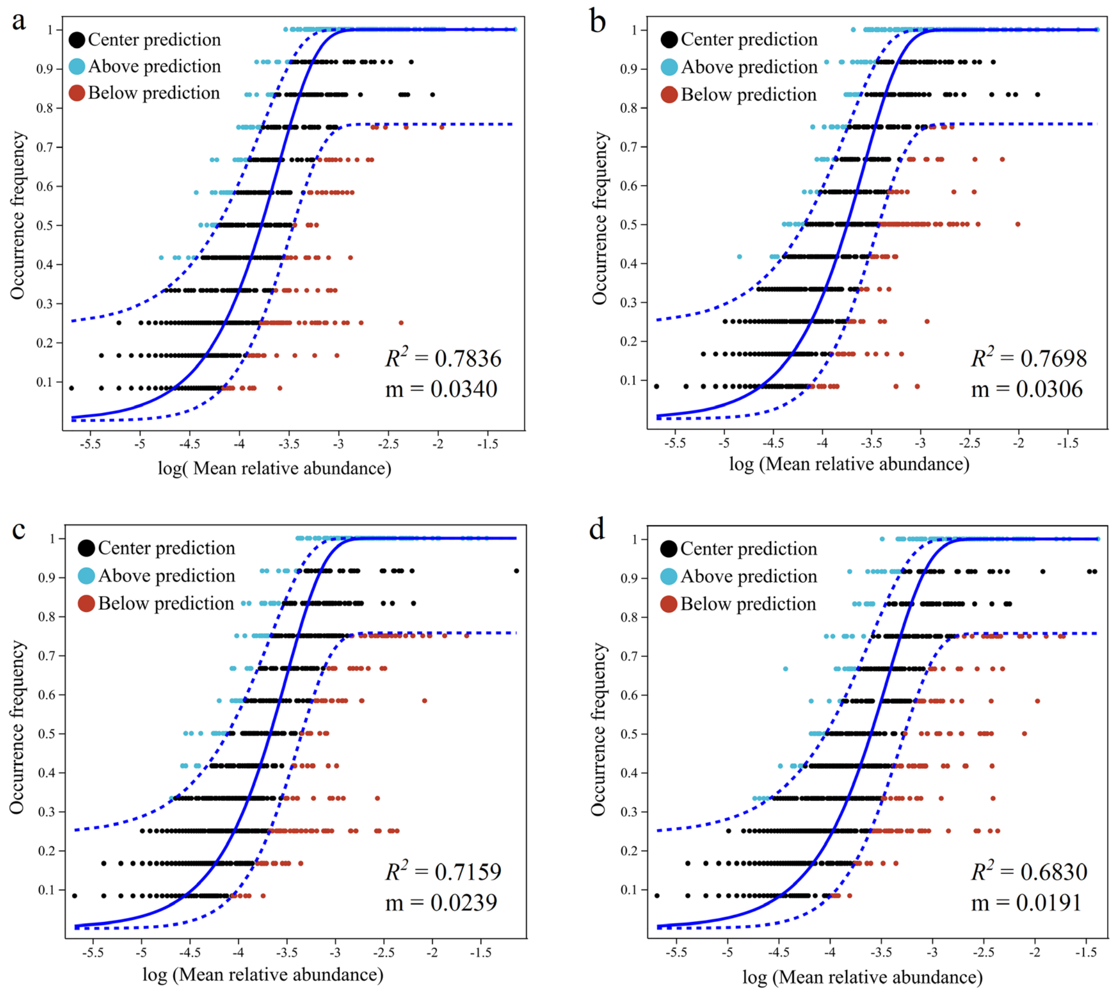


**Supplementary Fig. 9 | Neutral community model analysis of bacterial community assembly in V-carrier biofilms across seasonal temperature variations. a–d,** Fit of the neutral community model to observed ASV distributions in aerobic V-carrier biofilms sampled on **(a)** day 45 (16.3 °C), **(b)** day 156 (28.9 °C), **(c)** day 307 (9.7 °C), and **(d)** day 455 (22.7 °C). Each panel shows the relationship between mean relative abundance (log-transformed) and occurrence frequency. Solid curves represent the model's central prediction; upper and lower dashed curves indicate the 95% prediction interval. Points represent individual ASVs colored by their placement relative to the model prediction: blue (within prediction interval), orange (above prediction), and gray (below prediction). The coefficient of determination ($R^2$) and immigration rate (m) are indicated for each fit. The strong model fits ($R^2 > 0.68$ across all time points) demonstrate that stochastic processes (ecological drift, random dispersal) dominated bacterial community assembly in the V-carrier biofilms throughout the operational period, regardless of temperature fluctuations. This consistent neutral pattern highlights the role of carrier topology in creating a stable microenvironment that minimizes deterministic selection pressures.

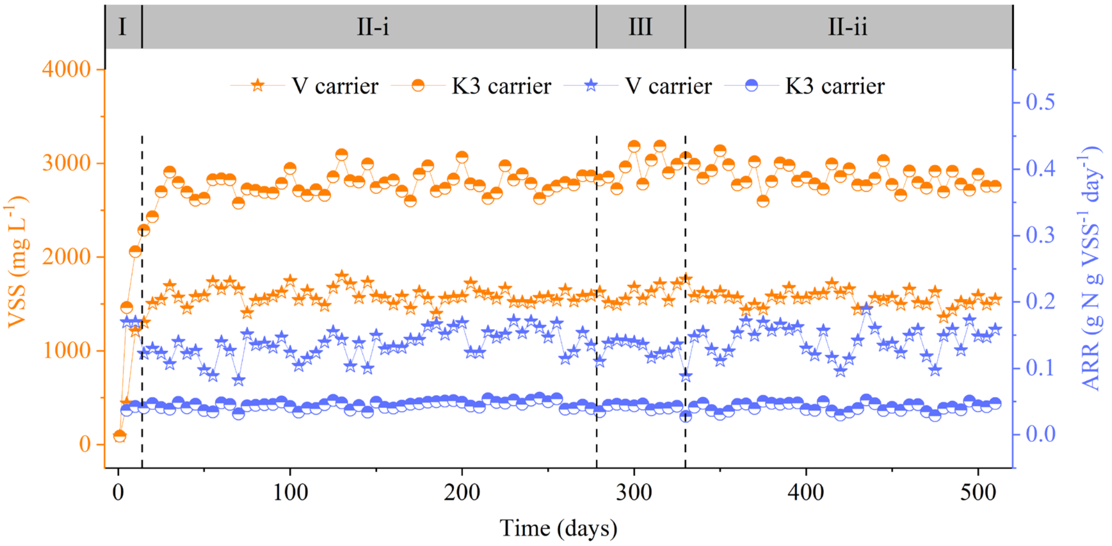


**Supplementary Fig. 10 | Comparative long-term performance of V-carrier and K3-carrier systems.** Temporal profiles of volatile suspended solids (VSS) concentrations in aerobic reactor biofilms for V-carrier (orange stars) and K3-carrier (orange circles) systems. Corresponding ammonia removal rates (ARR) for both carrier systems (V-carrier: blue stars; K3-carrier: blue circles). Operational phases are indicated: Phase I (start-up, days 1–10), Phase II-i (suitable temperature, days 11–280), Phase III (low temperature, days 281–324), and Phase II-ii (suitable temperature, days 325–512). The V-carrier system maintained significantly lower biofilm biomass (average VSS: ~1,569 mg $L^{-1}$) while achieving a 3.2-fold higher ammonia removal rate compared to the K3-carrier system (average VSS: ~2,807 mg $L^{-1}$), demonstrating more efficient biomass utilization. This biomass-decoupled performance highlights the advantage of the hydro-topological design for maintaining optimal mass transfer efficiency within a low-biomass system.

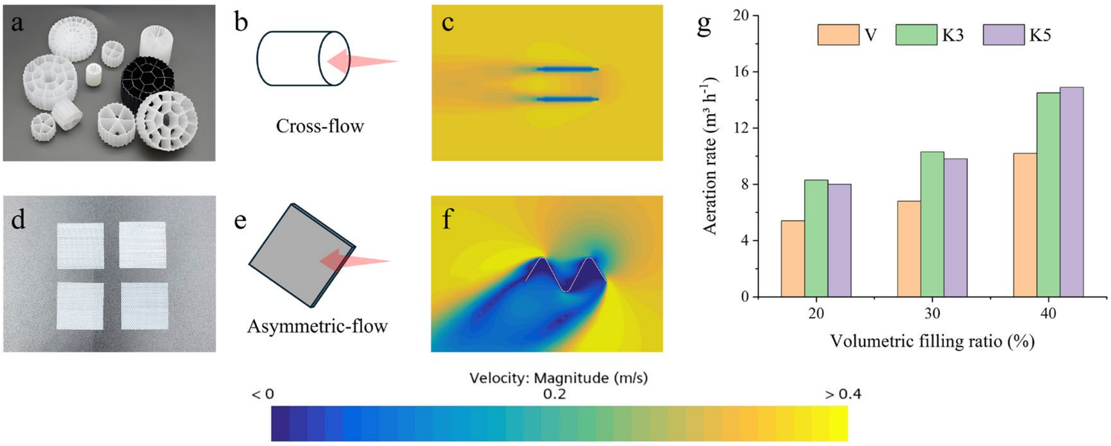


**Supplementary Fig. 11 | Hydrodynamic behavior and fluidization performance in pilot-scale reactors. a,** Overall view of conventional cylindrical carriers. **b-c,** Flow pattern around a cylindrical carrier (**b**) and the corresponding CFD-simulated flow field (**c**). **d,** Overall view of the V-carriers. **e-f,** Flow pattern around the square-shaped V-carrier (**e**) and the corresponding CFD-simulated flow field (**f**). **g,** Quantitative assessment of aeration intensity required for the square-shaped V-carrier and the cylindrical carriers (K3 and K5). Fluidization tests were conducted in a 2 $m^3$ pilot-scale bioreactor.

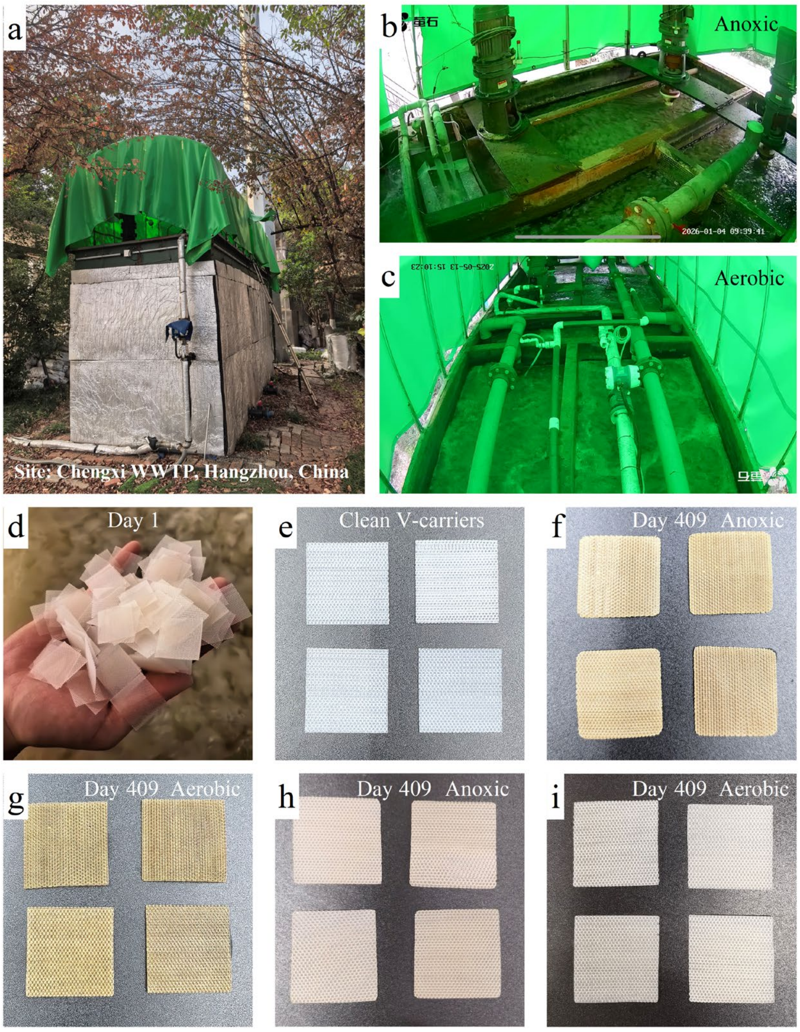


**Supplementary Fig. 12 | Pilot plant of the anoxic/aerobic-moving bed biofilm reactor (A/O-MBBR) process and overall morphology of V-carriers after long-term operation. a,** Installation site and overall view of the pilot plant. **b-c,** Field views of the anoxic (**b**) and aerobic (**c**) reactors, respectively. **d,** Carriers during complete fluidization. **e,** Clean and dried V-carriers. **f-g,** Morphology of biofilm-attached anoxic (**f**) and aerobic (**g**) carriers after 409 days of operation. **h-i,** Morphology of biofilm-detached anoxic (**h**) and aerobic (**i**) carriers after 409 days of operation. The pilot system was commissioned on November 12, 2024, and remains operational. It has a total working volume of 24 $m^3$, comprising a 9 $m^3$ anoxic reactor and a 15 $m^3$ aerobic reactor. The volumetric filling ratios of carriers were maintained at 16% and 30% in the anoxic and aerobic reactors, respectively. It operates in a pure biofilm mode (no sludge recirculation) with a 300% internal nitrified liquor recycle ratio. The system treats municipal wastewater with influent characteristics similar to those used in this study (see Supplementary Table 3 for concentrations). Operating at a 9-hour hydraulic retention time, it achieves nitrification and denitrification rates comparable to the laboratory-scale results. No carrier clogging has been observed, and the operational stability of the system has been fully validated.

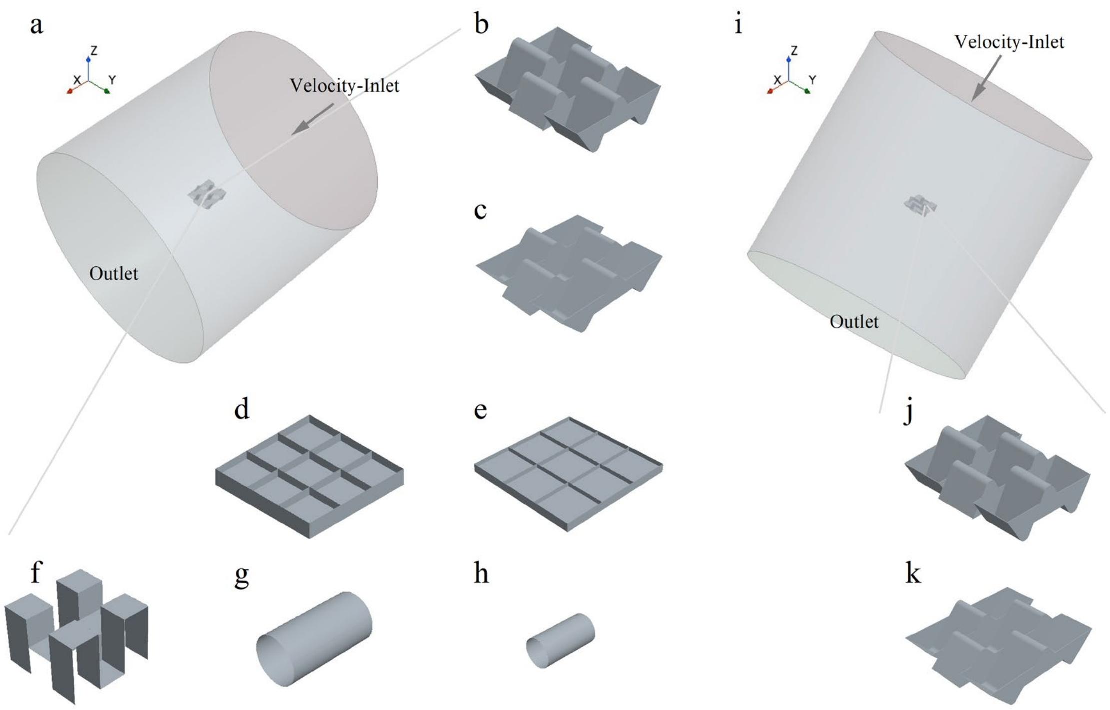


**Supplementary Fig. 13 | Computational fluid dynamics (CFD) simulations of the flow fields on carrier surfaces.** Panels (**a**) and (**i**) show the geometrical boundary conditions of the CFD model for parallel and 45° flow incidence, respectively. Further details are provided in Supplementary Note 7. **b–h, j, k,** Three-dimensional digital reconstructions of the trimmed scaffolds for the following carriers: (**b**, **j**) V-carrier with an opening ratio of 1.39 (1.0/0.72); (**c**, **k**) V-carrier with an opening ratio of 2.08 (1.5/0.72); (**d**) MK5 carrier with cell dimensions of 2.3 × 2.3 mm and a ridge height of 1.0 mm; (**e**) MK5 carrier with cell dimensions of 2.3 × 2.3 mm and a ridge height of 0.5 mm; (**f**) U-carrier with cell dimensions of 2.0 × 2.0 mm and a ridge height of 4.0 mm; (**g**) K3 carrier with a hollow diameter of 5 mm and a length of 10 mm; (**h**) K5 carrier with a hollow diameter of 2 mm and a length of 4 mm.

**References:**


1. Bassin, J. P., Kleerebezem, R., Rosado, A. S., van Loosdrecht, M. C. M. & Dezotti, M. Effect of Different Operational Conditions on Biofilm Development, Nitrification, and Nitrifying Microbial Population in Moving-Bed Biofilm Reactors. *Environ. Sci. Technol.* **46**, 1546-1555 (2012).
2. Yu, Y., Lee, C., Kim, J. & Hwang, S. Group-specific primer and probe sets to detect methanogenic communities using quantitative real-time polymerase chain reaction. *Biotechnol. Bioeng*. **89**, 670-679 (2005).
3. Dan, Q., Zhang, Q., Wang, T., Wang, H. & Peng, Y. Floc management enables integrated anammox and enhanced biological phosphorus removal for sustainable ultra-efficient nutrient removal. *Nature Water*. **3**, 201-210 (2025).
4. Campos Marin, A., Grossi, T., Bianchi, E., Dubini, G. & Lacroix, D. 2D μ-Particle Image Velocimetry and Computational Fluid Dynamics Study Within a 3D Porous Scaffold. *Ann. Biomed. Eng*. **45**, 1341-1351 (2017).
5. Xie, Y., Wan, A. & Wu, Y. Numerical simulation and experimental investigation on the effect of a new suspended carrier filler on mass transfer in MBBR. *Chemical Engineering and Processing - Process Intensification*. **156**, 108104 (2020).
6. M. Meky, M., Ali, M. N., Shaltout, F. & Danial, M. Numerical investigation of hydraulic characteristics in MBBR Systems: Comparative analysis of different Kaldnes K1 media configurations. *Bioresource Technology Reports*. **31**, 102220 (2025).
7. Ku, M. J. H. et al. Imaging viscous flow of the Dirac fluid in graphene. *Nature*. **583**, 537-541 (2020).
8. Ferris, M. J., Muyzer, G. & Ward, D. M. Denaturing gradient gel electrophoresis profiles of 16S rRNA-defined populations inhabiting a hot spring microbial mat community. *Appl. Environ. Microbiol*. **62**, 340-346 (1996).
9. Lane, D. J. 16S/23S r-RNA sequencing. In Nucleic acid techniques in bacterial systematics, Goodfellow, E. S. a. M., Ed. John Willey and Sons: New York, 1991; pp 115-175.
10. Tsushima, I., Kindaichi, T. & Okabe, S. Quantification of anaerobic ammonium-oxidizing bacteria in enrichment cultures by real-time PCR. *Water Res*. **41**, 785-794 (2007).
11. Kowalchuk, G. A. et al. Analysis of ammonia-oxidizing bacteria of the beta subdivision of the class Proteobacteria in coastal sand dunes by denaturing gradient gel electrophoresis and sequencing of PCR-amplified 16S ribosomal DNA fragments. *Appl. Environ. Microbiol*. **63**, 1489-1497 (1997).
12. Hermansson, A. & Lindgren, P. E. Quantification of ammonia-oxidizing bacteria in arable soil by real-time PCR. *Appl. Environ. Microbiol*. **67**, 972-976 (2001).
13. Dionisi, H. M. et al. Quantification of *Nitrosomonas oligotropha*-like ammonia-oxidizing bacteria and *Nitrospira* spp. from full-scale wastewater treatment plants by competitive

PCR. *Appl. Environ. Microbiol*. **68**, 245-253 (2002).
