# Supplementary material for: A hydro-topological strategy enables self-regulating biofilms for sustainable wastewater treatment
Source: Nat Commun. 2026 Mar 13;17:3878. doi: 10.1038/s41467-026-70682-y (PMC13125250; doi:10.1038/s41467-026-70682-y)
Supplement: Supplementary file 2 — Reporting Summary [file 41467_2026_70682_MOESM2_ESM.pdf]

Corresponding author(s): Ying Liu & Kuichuan Sheng

Last updated by author(s): Feb 16, 2026

# Reporting Summary

Nature Portfolio wishes to improve the reproducibility of the work that we publish. This form provides structure for consistency and transparency in reporting. For further information on Nature Portfolio policies, see our Editorial Policies and the Editorial Policy Checklist.

## Statistics

For all statistical analyses, confirm that the following items are present in the figure legend, table legend, main text, or Methods section.

| n/a | Confirmed | |
|---|---|---|
| ☐ | ☒ | The exact sample size (*n*) for each experimental group/condition, given as a discrete number and unit of measurement |
| ☐ | ☒ | A statement on whether measurements were taken from distinct samples or whether the same sample was measured repeatedly |
| ☐ | ☒ | The statistical test(s) used AND whether they are one- or two-sided<br>*Only common tests should be described solely by name; describe more complex techniques in the Methods section.* |
| ☒ | ☐ | A description of all covariates tested |
| ☒ | ☐ | A description of any assumptions or corrections, such as tests of normality and adjustment for multiple comparisons |
| ☐ | ☒ | A full description of the statistical parameters including central tendency (e.g. means) or other basic estimates (e.g. regression coefficient) AND variation (e.g. standard deviation) or associated estimates of uncertainty (e.g. confidence intervals) |
| ☐ | ☒ | For null hypothesis testing, the test statistic (e.g. *F*, *t*, *r*) with confidence intervals, effect sizes, degrees of freedom and *P* value noted<br>*Give P values as exact values whenever suitable.* |
| ☒ | ☐ | For Bayesian analysis, information on the choice of priors and Markov chain Monte Carlo settings |
| ☒ | ☐ | For hierarchical and complex designs, identification of the appropriate level for tests and full reporting of outcomes |
| ☐ | ☒ | Estimates of effect sizes (e.g. Cohen's *d*, Pearson's *r*), indicating how they were calculated |

*Our web collection on statistics for biologists contains articles on many of the points above.*

## Software and code

Policy information about availability of computer code

| | |
|---|---|
| Data collection | N/A |
| Data analysis | Statistical analyses were performed using OriginLab 2021 (OriginPro, OriginLab Corporation, Northampton, MA, USA) and Microsoft Excel 2021 (Microsoft Corporation, Redmond, WA, USA). Microbiological data were analyzed on the Majorbio Cloud Platform (https://cloud.majorbio.com). Experimental data are presented as mean ± standard deviation (s.d.). Specific statistical tests applied included the two-tailed paired t-test, Welch's two-tailed t-test, two-tailed Pearson correlation, and the two-tailed Adonis test. A P value of less than 0.05 was considered statistically significant. Data points below the detection limit were assigned a value of zero for analysis purposes. The significance level (α) was set at 0.05 for all tests. No data were excluded from the analyses unless otherwise specified. |

For manuscripts utilizing custom algorithms or software that are central to the research but not yet described in published literature, software must be made available to editors and reviewers. We strongly encourage code deposition in a community repository (e.g. GitHub). See the Nature Portfolio guidelines for submitting code & software for further information.

## Data

Policy information about availability of data

All manuscripts must include a data availability statement. This statement should provide the following information, where applicable:

- Accession codes, unique identifiers, or web links for publicly available datasets
- A description of any restrictions on data availability
- For clinical datasets or third party data, please ensure that the statement adheres to our policy

The data generated in this study are provided within the article and its Supplementary Information. Raw sequence data have been deposited in the China National Center for Bioinformation (CNCB) database under BioProject accession number CRA032524. Source data are provided with this paper.

## Research involving human participants, their data, or biological material

Policy information about studies with human participants or human data. See also policy information about sex, gender (identity/presentation), and sexual orientation and race, ethnicity and racism.

| | |
|---|---|
| Reporting on sex and gender | N/A |
| Reporting on race, ethnicity, or other socially relevant groupings | N/A |
| Population characteristics | N/A |
| Recruitment | N/A |
| Ethics oversight | N/A |

Note that full information on the approval of the study protocol must also be provided in the manuscript.

# Field-specific reporting

Please select the one below that is the best fit for your research. If you are not sure, read the appropriate sections before making your selection.

☐ Life sciences ☐ Behavioural & social sciences ☒ Ecological, evolutionary & environmental sciences

For a reference copy of the document with all sections, see nature.com/documents/nr-reporting-summary-flat.pdf

# Ecological, evolutionary & environmental sciences study design

All studies must disclose on these points even when the disclosure is negative.

| | |
|---|---|
| Study description | This study presents a hydro-topological design strategy for moving bed biofilm reactor (MBBR) carriers that enables self-regulating biofilms for sustainable wastewater treatment. Through an integrated approach combining long-term reactor operation (> 500 days), microbial community analysis, and computational fluid dynamics (CFD) simulations, we demonstrate that the V-shaped carrier design achieves superior biofilm control and nutrient removal performance compared to conventional carriers. The research establishes a new paradigm where controlled hydraulic exposure, rather than geometric confinement alone, governs biofilm self-regulation and system stability. |
| Research sample | The study employed multiple research samples, including six distinct MBBR carrier types (V-carrier, K3, K5, U-carrier, MK5, and Mutagbiochip) that were tested. Biofilm samples were collected at strategic time points over more than 500 days of operation. Municipal wastewater from the septic tank effluent of Shangkun Park (Hangzhou, China) was used as the treatment substrate. |
| Sampling strategy | The sampling strategy employed a systematic, multi-scale approach to evaluate carrier performance and system functionality throughout the 500-day operational period. Daily influent and effluent samples were collected for water quality analysis. Biofilm samples were periodically harvested from carriers at strategic time points, with samples preserved at –80 °C for subsequent physicochemical and microbiological characterization. To assess functional activity, 300 V-carriers were aseptically retrieved every two days for ex situ batch assays. This integrated strategy facilitated comprehensive data collection across temporal and biological scales. |
| Data collection | Wastewater and biofilm samples were collected and analyzed by the authors working at Tao of Water Research Institute. Daily influent and effluent samples were filtered through 0.45-µm membranes for immediate spectrophotometric analysis of sCOD, $NH_4^+$-N, TIN, and $PO_4^{3-}$-P concentrations. Biofilm samples were harvested from carriers via gentle brushing or chemical detachment, with subsamples either immediately analyzed for SS/VSS, elemental composition (via ICP-MS), and morphological characteristics, or preserved at -80°C for subsequent DNA extraction. Microbiological analyses included qPCR quantification of functional genes, 16S rRNA amplicon sequencing performed on an Illumina MiSeq platform, FISH-CLSM for spatial distribution analysis, and live-dead staining for viability assessment. Ex situ batch assays were conducted every two days using 300 aseptically retrieved carriers to |

| | |
|---|---|
| | determine temperature-dependent nitrification potential under controlled conditions. All analytical procedures followed standardized protocols with appropriate quality controls, including triplicate measurements for molecular analyses and parallel control assays for abiotic verification. |
| Timing and spatial scale | The study was conducted over 500 days (March 11, 2023, to August 15, 2024) at the Tao of Water Research Institute in Hangzhou, China. The system treated raw municipal wastewater collected daily from the site's septic tank, providing a realistic operational context for evaluating long-term biofilm performance. |
| Data exclusions | No data were excluded from the analyses. |
| Reproducibility | All wastewater analyses were conducted in accordance with the standard methods prescribed by the American Public Health Association, and all measurements were performed in triplicate to ensure reproducibility. |
| Randomization | N/A |
| Blinding | N/A |

Did the study involve field work? ☒ Yes ☐ No

## Field work, collection and transport

| | |
|---|---|
| Field conditions | Laboratory-scale anoxic/aerobic moving bed biofilm reactor systems were operated at the Tao of Water Research Institute in Hangzhou, China (30.32°N, 120.07°E), a south temperate zone location experiencing natural seasonal temperature variations from 34.0°C to -2.0°C. These conditions provided a realistic environment for evaluating biofilm carrier performance under dynamic thermal fluctuations. |
| Location | The laboratory-scale anoxic/aerobic (A/O) moving bed biofilm reactor (MBBR) systems were operated at the Tao of Water Research Institute in Hangzhou, China (30.32°N, 120.07°E). |
| Access & import/export | N/A |
| Disturbance | N/A |

# Reporting for specific materials, systems and methods

We require information from authors about some types of materials, experimental systems and methods used in many studies. Here, indicate whether each material, system or method listed is relevant to your study. If you are not sure if a list item applies to your research, read the appropriate section before selecting a response.

### Materials & experimental systems

| n/a | Involved in the study | |
|---|---|---|
| ☒ | ☐ | Antibodies |
| ☒ | ☐ | Eukaryotic cell lines |
| ☒ | ☐ | Palaeontology and archaeology |
| ☒ | ☐ | Animals and other organisms |
| ☒ | ☐ | Clinical data |
| ☒ | ☐ | Dual use research of concern |
| ☒ | ☐ | Plants |

### Methods

| n/a | Involved in the study | |
|---|---|---|
| ☒ | ☐ | ChIP-seq |
| ☒ | ☐ | Flow cytometry |
| ☒ | ☐ | MRI-based neuroimaging |

## Plants

| | |
|---|---|
| Seed stocks | *Report on the source of all seed stocks or other plant material used. If applicable, state the seed stock centre and catalogue number. If plant specimens were collected from the field, describe the collection location, date and sampling procedures.* |
| Novel plant genotypes | *Describe the methods by which all novel plant genotypes were produced. This includes those generated by transgenic approaches, gene editing, chemical/radiation-based mutagenesis and hybridization. For transgenic lines, describe the transformation method, the number of independent lines analyzed and the generation upon which experiments were performed. For gene-edited lines, describe the editor used, the endogenous sequence targeted for editing, the targeting guide RNA sequence (if applicable) and how the editor was applied.* |
| Authentication | *Describe any authentication procedures for each seed stock used or novel genotype generated. Describe any experiments used to assess the effect of a mutation and, where applicable, how potential secondary effects (e.g. second site T-DNA insertions, mosiacism, off-target gene editing) were examined.* |
